# Abiraterone vs Enzalutamide Among US Veterans With Metastatic Hormone-Sensitive Prostate Cancer

**DOI:** 10.1001/jamanetworkopen.2025.40730

**Published:** 2025-11-04

**Authors:** Harshraj Leuva, Mengxi Zhou, Benjamin A. Teply, Yeun-Hee Anna Park, Carol Luhrs, Prabhjot Singh Mundi, Susan E. Bates, Izak Faiena, Tito Fojo, Martin W. Schoen

**Affiliations:** 1 University of Nebraska Medical Center, Omaha; 2 Columbia University Herbert Irving Comprehensive Cancer Center, New York, New York; 3 SUNY Downstate Health Sciences University, Brooklyn, New York; 4 James J. Peters Bronx Veterans Affairs Medical Center, Bronx, New York; 5 Veterans Affairs New York Harbor Healthcare System – Brooklyn Campus; 6 Saint Louis Veterans Affairs Medical Center, St Louis, Missouri; 7 Saint Louis University School of Medicine, St Louis, Missouri

## Abstract

**Importance:**

The choice of first line hormonal therapy in metastatic hormone-sensitive prostate cancer (mHSPC) is often based on comorbidities or physician preference due to the lack of data comparing abiraterone and enzalutamide in clinical trials and in clinical practice settings, especially in African American patients.

**Objective:**

To compare clinical outcomes in patients with mHSPC treated with abiraterone acetate or enzalutamide.

**Design, Setting, and Participants:**

This cohort study included all patients with mHSPC diagnoses in the US Veterans Affairs Health Care system who initiated abiraterone or enzalutamide between July 2017 and April 2023. Data were analyzed from April 2024 to March 2025.

**Exposures:**

Receipt of abiraterone acetate or enzalutamide.

**Main outcomes and measures:**

Drug outcomes were established by estimating rates of tumor growth (*g*-rates) using prostate-specific antigen (PSA) values while receiving therapy and measuring overall survival (OS). Two different matching analyses were performed: inverse probability weighting (IPW) and 1:1 exact matching.

**Results:**

A total of 1258 patients (median [IQR] age, 73 [69-79] years; 314 [25.0%] African American, 857 [68.1%] White, and 87 [6.9%] with other or unknown race) who received abiraterone and 311 patients (median [IQR] age, 74 [69-79] years; 84 [27.0%] African American, 207 [66.6%] White, and 20 [6.4%] with other or unknown race) who received enzalutamide were included. In the unweighted analysis, the abiraterone cohort had median (IQR) *g*-rate of 0.000137/d (0.000094-0.001519) and the enzalutamide cohort, 0.000137/d (0.000098-0.001815); median (IQR) OS was 36.2 (32.8-38.8) months for abiraterone and 36.2 (34.1-40.5) months for enzalutamide, with a median (IQR) follow-up of 28.7 (15.6-45.6) and 30.8 (16.1-39.1) months, respectively. In IPW analysis, using abiraterone as reference, the weighted median OS was comparable between abiraterone and enzalutamide in the full cohort (36.2; 95% CI, 32.8-38.8 vs 35.5; 95 % CI, 32.9-40.4 months; hazard ratio [HR], 1.09; 95% CI, 0.92-1.30; *P* = .32), African American veterans (39.7; 95% CI, 34.3-46.6 vs 40.3; 95% CI, 34.3-not reached months; HR, 0.98; 95% CI, 0.72-1.34; *P* = .90) and those with cardiovascular disease (31.5; 95% CI, 28.1-35.5 vs 35.0; 95% CI, 30.7-38.9; HR, 1.12; 95% CI, 0.91-1.37; *P* = .30). In 1:1 matched analysis, both the abiraterone and enzalutamide groups had 279 patients with 63 (23%) who were African American; these patients had a median (IQR) follow-up of 27.3 (15.3-40.8) and 31.1 (16.8-39.9) months, respectively. The 1:1 matched cohort consisted of 158 patients (57%) in each group with an initial Gleason score of 8 or higher, and 224 patients (80%) in each group with a PSA value of 50 ng/mL or higher at treatment initiation. For the 1:1 matched cohort and its subgroups of African American patients, patients with Gleason scores of 8 or higher, and patients with a starting PSA level of 50 ng/mL or higher, no statistically significant difference in median *g*-rate and OS was observed.

**Conclusions and Relevance:**

This cohort study found that abiraterone and enzalutamide had comparable outcomes in a first-line mHSPC setting, with similar *g*-rates and OS in both White and African American patients. There were no differences in survival or *g*-rate between treatment based on race, comorbidities, and prostate cancer features, including Gleason score, PSA, and volume of disease.

## Key Points

**Question:**

Do abiraterone and enzalutamide have comparable outcomes in patients with metastatic hormone-sensitive prostate cancer?

**Findings:**

In this cohort study using data from 1569 US veterans, those receiving abiraterone and enzalutamide had similar estimated rates of tumor growth and overall survival. These findings did not change after performing either inverse probability weighting or 1:1 exact matching.

**Meaning:**

These findings suggest abiraterone and enzalutamide have comparable outcomes in patients with metastatic hormone-sensitive prostate cancer.

## Teaser

This cohort study evaluates clinical outcomes in patients with metastatic hormone-sensitive prostate cancer treated with abiraterone acetate or enzalutamide.

## Introduction

Metastatic prostate cancer (mPC) is initially sensitive to hormonal blockade and is either named metastatic hormone-sensitive prostate cancer (mHSPC) or metastatic castration-sensitive prostate cancer (mCSPC). mHSPC includes patients who present with de novo metastatic disease or develop recurrent disease after initial definitive surgical or radiation treatment and have testosterone levels higher than 50 ng/dL (to convert to nanomoles per liter, multiply by 0.0347).^1^ While often viewed as an indolent cancer, mPC has a cancer-specific mortality rate of 78% and a 5-year overall survival rate of only 34%.^2,3^ Additionally, prostate cancer mortality rates in African American men are approximately 2 to 4 times higher than those in every other racial and ethnic group.^4,5^

Recent advances in the therapy of mHSPC have led to significantly improved overall survival (OS) during the last decade in patients with mHSPC in clinical trials and the general US population.^6^ Progress began with the Chemohormonal Therapy Versus Androgen Ablation Randomized Trial for Extensive Disease in Prostate Cancer trial that reported improvement in OS with the addition of 6 cycles of docetaxel to androgen deprivation therapy (ADT), especially in patients with high-volume metastatic disease.^7,8^ Between 2018 and 2022, 4 novel hormonal therapies or androgen receptor pathway inhibitors (NHT/ARPIs)—abiraterone, enzalutamide, darolutamide, and apalutamide—received regulatory approvals for the treatment of mHSPC in combination with ADT, based on data from their registration studies.^9,10,11,12^ The abiraterone, enzalutamide, and apalutamide studies compared a combination of an ARPI plus ADT with ADT alone, and the Darolutamide in Addition to Standard Androgen Deprivation Therapy and Docetaxel in Metastatic Hormone-Sensitive Prostate Cancer (ARASENS) study compared an ARPI plus docetaxel plus ADT with docetaxel plus ADT.

There has been an increase in the use of ARPIs for mHSPC, with 53.7% of veterans receiving ARPIs in 2022.^13^ However, there have been no direct randomized trials comparing ARPIs in hormone-sensitive disease. The choice of drug is typically based on the patient’s comorbidities, potential interactions with other medications, and the prescribing physician’s familiarity with the medication. Additionally, data regarding the efficacy of these medications in African American men is limited. The LATTITUDE trial^9^ did not provide patient numbers based on race, while the ARCHES,^10^ TITAN,^11^ and ARASENS^12^ trials enrolled 8 (1.4%), 10 (1.9%), and 26 (4.3%) African American men, respectively.

The US Veterans Health Administration (VHA), the nation’s largest integrated health care system, provides more equal care for veterans, mitigating the impact of socioeconomic factors. The VHA pharmacy provides all drugs to veterans, minimizing barriers to treatment due to cost and insurance. All veteran data are securely stored in the Department of Veterans Affairs (VA) Corporate Data Warehouse (CDW) and are accessible to researchers through the VA Informatics and Computing Infrastructure (VINCI). Unstructured data are updated daily, while structured data are updated monthly. Since 2015, there have been more than 40 000 veterans with mPC receiving therapies throughout their disease course in the VHA. Additionally, there are over 8000 African American veterans treated for mPC in the VHA, creating a more diverse population that can be used to study treatment outcomes.

While clinical trials usually assess both prostate-specific antigen (PSA) and imaging end points, the PSA trajectory typically guides and informs disease progression in clinical practice settings. In the case of mPC, where most interventions are not curative but palliative, the trajectory of PSA values is influenced by the concomitant occurrence of 2 processes—regression of the treatment-sensitive tumor fraction and growth of the treatment-resistant disease. Despite their simultaneous occurrence, the concomitant rates of regression or decay of the treatment-sensitive fraction and growth of the treatment-resistant component can be calculated with simple mathematical formulas. We have developed an approach that allows us to estimate these concomitant rates and report the outcomes as the rate of growth of the treatment-resistant fraction, a value we designate as the *g*-rate. This value can be determined using imaging measurements or any other measure of tumor burden, such as PSA values for patients with prostate cancer.^14,15,16^ Since tumors that decrease do not cause mortality, the rate at which the treatment-sensitive fraction that will be eliminated regresses or decays (*d*) has no impact on overall survival.

We have identified robust inverse correlations between *g*-rates generated using serial on-treatment PSA values and OS in prostate cancer and *g*-rates generated using serial on-treatment imaging across a spectrum of other solid tumors treated with diverse therapeutic approaches such as hormonal therapies, chemotherapy, targeted therapies, and immunotherapy.^14,15,16,17,18,19,20^ The US Food and Drug Administration (FDA) has also confirmed this correlation in an independent analysis.^21^ We have previously reported the outcomes of abiraterone and enzalutamide in patients with metastatic castration-resistant prostate cancer using our VA database and *g*-rate method.^15^ In the current observational study, we compare the outcomes of abiraterone and enzalutamide in patients with mHSPC using this approach and report on their survival outcomes.

## Methods

Eligible patients with mHSPC were identified in the VA CDW using VA VINCI to identify veterans with mPC based on *International Classification of Diseases, Ninth Revision (ICD-9)* and *ICD-10* codes. The data are then cross-referenced with the CDW Oncology Registry (VACCR) and the VA Prostate Data Core to confirm the diagnosis.^22^ Oral and intravenous (IV) medication dispensing details were obtained from the CDW pharmacy database. This cohort study was reviewed and approved by the VA Bronx Health Care System institutional review board and was performed in accordance with the Declaration of Helsinki. A waiver of consent was approved due to use of deidentified data. The results are reported according to the Strengthening the Reporting of Observational Studies in Epidemiology (STROBE) reporting guideline.

### Patient Cohort

Veterans with mHSPC who received abiraterone or enzalutamide as the first line for mHSPC and had estimable rates of tumor growth (*g*-rate) from PSA values using the tumGr software package obtained while receiving the medications of interest were included in this cohort. All patients received ADT with luteinizing hormone–releasing hormone (LHRH) agonist or antagonist or LHRH antagonists. The mHSPC cohort was identified in 2 ways, either by meeting all 3 criteria of (1) starting therapy within 6 months of prostate cancer diagnosis with confirmed metastases, (2) having no PSA progression before medication start, and (3) receiving medications after publication of the clinical trial supporting its use for mHSPC^9,10^; or by having a testosterone value above 50 ng/dL in the preceding 90 days of starting the drug of interest. Additionally, these data were then cross-referenced with VACCR data. If the patient received either of these medications within 1 year of regional lymph node disease designation without any other metastatic site, they were excluded. Patients receiving concurrent docetaxel for mHSPC were excluded.

### Covariates

We extracted data regarding demographics (age and race [African American, White, or other, which included American Indian or Alaska Native, Asian, Native Hawaiian or Other Pacific Islander), Gleason scores, treatment setting (rural vs urban), subsequent lines of therapy, comorbidities (calculated Charlson Comorbidity Index [CCI] using the Quan algorithm), and laboratory results (PSA) while patients were receiving medication. Race was evaluated in this study because there have been limited data on race in clinical trials and there is concern of disparity of outcomes between different races in metastatic prostate cancer.

### *g*-Rate Method of Data Analysis Using Serial PSA Values While Recieving Therapy

The regression-growth models describe changes in tumor quantity during therapy resulting from simultaneous exponential decay or regression, termed *d*, and exponential growth or regrowth of the tumor, termed *g*. This basic mathematical model is:

f(*t*) = exp(−*d* × *t*) + exp(*g* × *t*) – 1.

At the time (*t*), the total tumor burden (*f*) is the sum of the therapy-sensitive part of the tumor regressing or decaying at the rate of decay (*d*) per day and the therapy-resistant part of the tumor growing exponentially at the rate of growth (*g*) per day.^23^

Both rates are calculated using the TUMGr package for R using serial PSA values while receiving a drug.^24^ This approach has been validated in patients with prostate cancer using clinical trial data and VINCI data.^14,15,16^ For this study, *g*-rate was calculated using all serial PSA values while the patients were receiving the drugs of interest. We also computed *g*-rate using serial PSA values available within the first 3 months (3-month *g*-rate) and first 6 months (6-month *g*-rate) of treatment. The doubling time (DT) can be readily calculated by dividing 0.693—the natural logarithm of 2—by the *g*-rate (DT = 0.693 / *g*-rate).^23^

### Outcome

On treatment PSA-based *g*-rate and OS were the primary outcomes of interest; death dates were collected from VA vital status files. OS times were estimated by determining the difference between the medication start date and either the death date or the last date of contact in the electronic medical record as of October 28, 2024.

### Inverse Probability Weighting

We performed multivariable inverse probability weighting (IPW) analysis to adjust for the outcomes of confounding variables. Variables included age, race, PSA at start of treatment, medication start year, and CCI excluding cancer diagnosis. The covariates were selected based on their known effects on OS in previous studies, in separate VA studies, or based on our prior observation of their effect in our dataset.^15,25^ We then created 4 different models incorporating various combinations of either numeric or continuous or categorical data inputs from these variables of interest. The categorical variables included age (<70, 70-74, 75-79, or ≥80 years), race, drug start year (≤2018 or ≥2020), starting PSA (<50 or ≥50 ng/mL; to convert to micrograms per liter, multiply by 1), CCI excluding cancer diagnosis (<5 or ≥5) and Gleason score (<8 or ≥8). The subgroup analysis of cardiovascular diseases included patients with myocardial infarction, heart failure, cerebrovascular disease, and peripheral vascular disease as noted by *ICD-9 *or *ICD-10* codes used to calculate the CCI.

### Exact Matching

**Figure 1.**
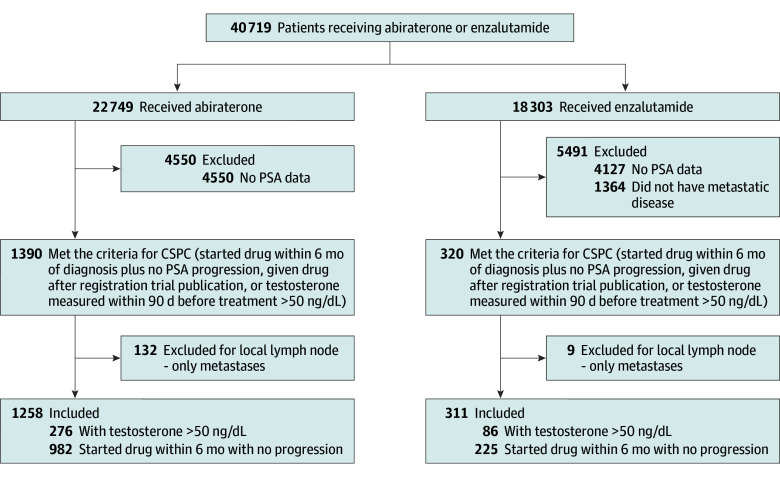
Study Flow Diagram CSPC indicates castration-sensitive prostate cancer; PSA, prostate-specific antigen.

Using VINCI data, we have developed a large reference cohort that includes 18 199 and 14 176 veterans who received abiraterone and enzalutamide, respectively, as of April 2023 and had at least 1 PSA value recorded (Figure 1). We created a 1:1 exact-matched cohort for patients receiving abiraterone or enzalutamide in mHSPC. Matching categorical variables included age (<70, 70-74, 75-79 or ≥80 years), race, drug start year (≤2018 or ≥2020), starting PSA (<50 or ≥50 ng/mL), CCI excluding cancer diagnosis (<5 or ≥5), and Gleason score (<8 or ≥8).

### Statistical Analysis

A log-rank test was used to compare the difference between the survival curves, and Cox proportional hazards modeling was performed to calculate the hazard ratio between treatments. Abiraterone was used as the reference for all hazard ratio calculations. A 2-sided *P* value less than .05 was considered statistically significant. For 1:1 exact matching, outcomes were analyzed using pair-stratified methods—conditional logistic regression for binary outcomes, stratified Cox regression for survival, and paired tests for continuous outcomes. All analyses were performed using R Statistical Software, version 4.2.0 (R Project for Statistical Computing) from April 2024 to March 2025.

## Results

We identified 1569 veterans treated for mHSPC, of which 1258 patients (80.2%; median [IQR] age, 73 [69-79] years; 314 [25.0%] African American, 857 [68.1%] White, and 87 [6.9%] with other or unknown race) received abiraterone and 311 patients (19.8%; median [IQR] age, 74 [69-79] years; 84 [27.0%] African American, 207 [66.6%] White, and 20 [6.4%] with other or unknown race) received enzalutamide as first-line therapy for mHSPC between July 2017 and April 2023 (Figure 1). In our cohort, 393 veterans (25.0%) were treated in rural setting, 402 (25.6%) had CCI scores of 5 or higher, and 855 (54.5%) had Gleason scores of 8 or higher. The median (range) PSA at the start of therapy was 8 (1-46) ng/mL for the cohort, with 300 (23.8%) and 73 (23.5%) veterans having starting PSA values of 50 ng/mL or higher with abiraterone and enzalutamide, respectively. There were no statistically significant differences between the abiraterone and enzalutamide groups for treatment setting (urban vs rural), CCI, Gleason scores, and PSA at the start of therapy (Table). The median (IQR) duration of treatment was 12.0 (5.4-24.0) months for abiraterone and 13.9 (5.7-22.7) months for enzalutamide, which did not differ statistically. The median (IQR) follow-up time was 28.7 (15.6-45.6) months for abiraterone and 30.8 (16.1-39.1) months for enzalutamide (Table).

**Table.** Baseline Characteristics

Characteristic	Patients, No. (%)		*P* value
	Abiraterone (n = 1258)	Enzalutamide (n = 311)	
Age, y			
Median (IQR)	73 (69-79)	74 (69-79)	.29
<70	364 (28.9)	79 (25.4)	.61
70-74	341 (27.1)	92 (29.6)	
75-79	257 (20.4)	67 (21.5)	
≥80	296 (23.5)	73 (23.5)	
Race			
African American	314 (25.0)	84 (27.0)	
White	857 (68.1)	207 (66.6)	.74
Other or unknown^a^	87 (6.9)	20 (6.4)	
Ethnicity			
Hispanic	55 (4.4)	11 (3.5)	.50
Not Hispanic	1122 (89.2)	275 (88.4)	
Unknown	81 (6.4)	25 (8.0)	
Rurality			
Urban	912 (72.5)	220 (70.7)	.74
Rural	310 (24.6)	83 (26.7)	
Unknown	36 (2.9)	8 (2.6)	
PSA, ng/mL			
At diagnosis, median (IQR)	39 (7-203)	36 (8-242)	.89
At treatment start, median (IQR)	8 (1-46)	8 (1-44)	.31
At treatment start group			
<50	958 (76.2)	238 (76.5)	.95
≥50	300 (23.8)	73 (23.5)	
Gleason score			
<8	151 (12.0)	41 (13.2)	.76
≥8	684 (54.4)	171 (55.0)	
Unknown	423 (33.6)	99 (31.8)	
Start year of treatment			
≤2018	136 (10.8)	14 (4.5)	<.001
≥2020	784 (62.3)	139 (44.7)	
2021 to present	338 (26.9)	158 (50.8)	
Treatment duration, d, median (IQR)	360 (162-720)	416 (171-680)	.97
Other prior treatment			
Surgery^b^	98 (7.8)	29 (9.3)	.44
Radiotherapy	602 (47.9)	129 (41.5)	.05
CCI without cancer			
<5	938 (74.6)	229 (73.6)	.79
≥5	320 (25.4)	82 (26.4)	
Comorbidities			
Myocardial infarction	202 (16.1)	49 (15.8)	.97
Heart failure	334 (26.6)	88 (28.3)	.58
Peripheral vascular disease	506 (40.2)	117 (37.6)	.44
Cerebrovascular disease	336 (26.7)	71 (22.8)	.19
Dementia	97 (7.7)	20 (6.4)	.52
Chronic pulmonary disease	466 (37.0)	126 (40.5)	.29
Rheumatic disease	157 (12.5)	38 (12.2)	.98
Peptic ulcer disease	47 (3.7)	14 (4.5)	.64
Mild liver disease	201 (16.0)	68 (21.9)	.02
Diabetes with or without complications	510 (40.5)	144 (46.3)	.07
Paraplegia and hemiplegia	73 (5.8)	10 (3.2)	.09
Kidney disease	396 (31.5)	107 (34.4)	.36
Moderate or severe liver disease	11 (0.9)	4 (1.3)	.51
HIV	11 (0.9)	0 (0)	.14
Median (IQR) follow-up in October 2024, mo	28.7 (15.6-45.6)	30.8 (16.1-39.1)	.12

Abbreviations: CCI, Charlson Comorbidity Index; PSA, prostate-specific antigen.

SI conversion factor: To convert PSA to micrograms per liter, multiply by 1.

^a^ Other includes American Indian or Alaska Native, Asian, Native Hawaiian or Other Pacific Islander.

^b^ Surgery includes prostatectomy and orchiectomy.

**Figure 2.**
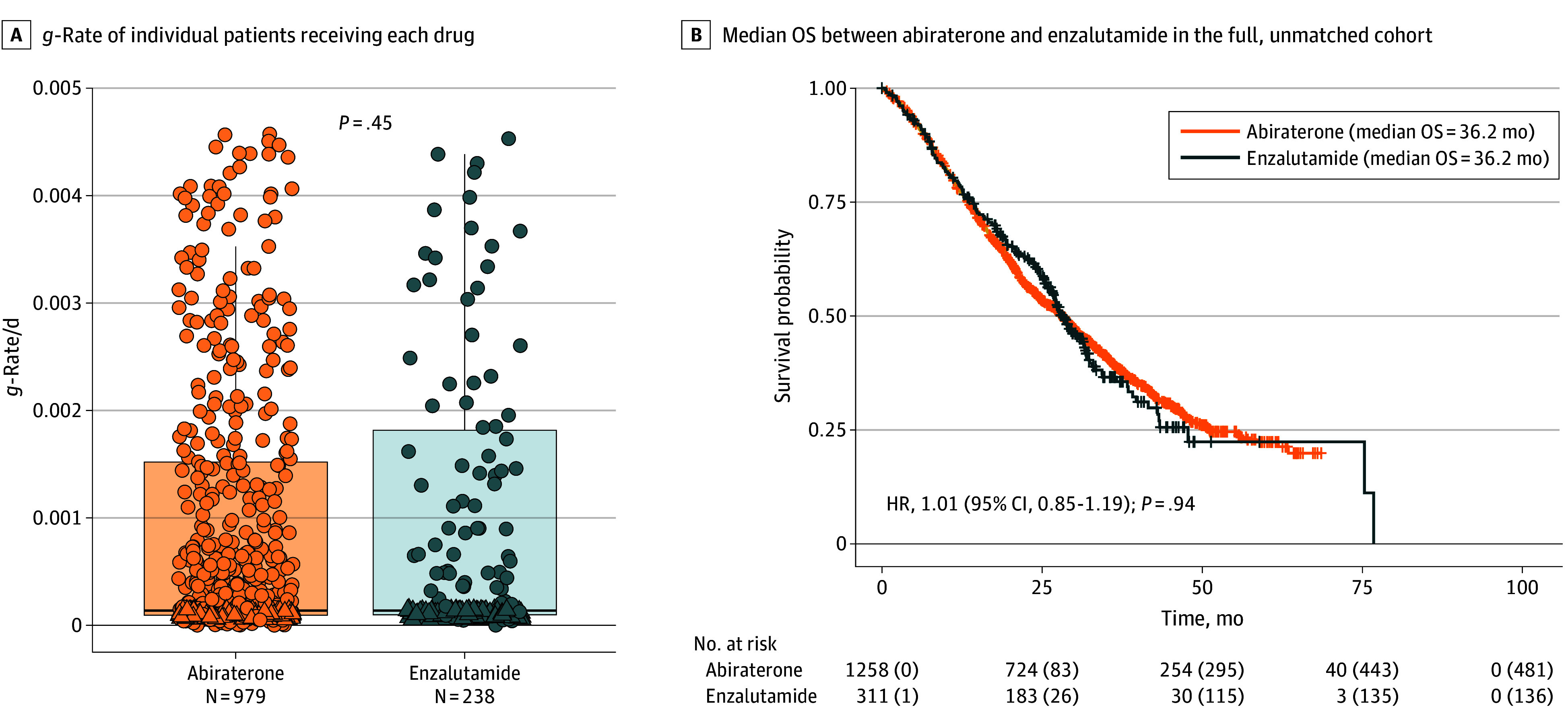
Comparison of Growth Rate (*g*-Rate) and Median Overall Survival (OS) for Abiraterone vs Enzalutamide A, Comparison of *g*-rates of individual patients receiving each drug. Dots indicate individual data; box, IQR; line, median; and whiskers, data that fall within 1.5 times the IQR. Dots outside of whiskers indicate outliers. B, Kaplan-Meier analysis of overall survival between abiraterone and enzalutamide in the full, unmatched cohort. HR indicates hazard ratio.

The median (IQR) *g*-rate for abiraterone was 0.000137/d (0.000094-0.001519) (doubling time [DT], 5068 days). There was no statistically significant difference when compared with the median (IQR) *g*-rate of enzalutamide of 0.000137/d (0.000098-0.001815) (DT, 5069 days) (Figure 2A). The median (IQR) OS was 36.2 (32.8-38.8) months for abiraterone and 36.2 (34.1-40.5) months for enzalutamide, with no significant difference (Figure 2B). Of the 1569 patients, 875 (55.7%) and 1097 (70%) veterans had calculable *g*-rates using available PSA values in the first 3 and 6 months of starting therapy, respectively (eTable 1 in Supplement 1). There was no statistically significant difference between abiraterone and enzalumide in both 3-month and 6-month median *g*-rate (eFigure 1 in Supplement 1).

We identified 765 veterans (48.8%) with information on the site of metastases and volume of disease. No significant differences in median *g*-rate or OS were observed between abiraterone and enzalutamide by disease volume (high vs low) or metastatic site (bone only vs visceral disease) (eFigures 2 and 3 in Supplement 1). Additional sensitivity analyses were conducted, which noted that patients’ characteristics of this subgroup of 765 patients were statistically different than the primary cohort with this subgroup of patients having higher median (range) PSA at treatment start of 11 (2-56) ng/mL, higher number of patients with Gleason scores of 8 or higher (455 patients [59%]), and more patients with CCI scores of 5 or higher (240 patients [31.4%]) (eTable 2 in Supplement 1).

### Inverse Probability Weighting Results

**Figure 3.**
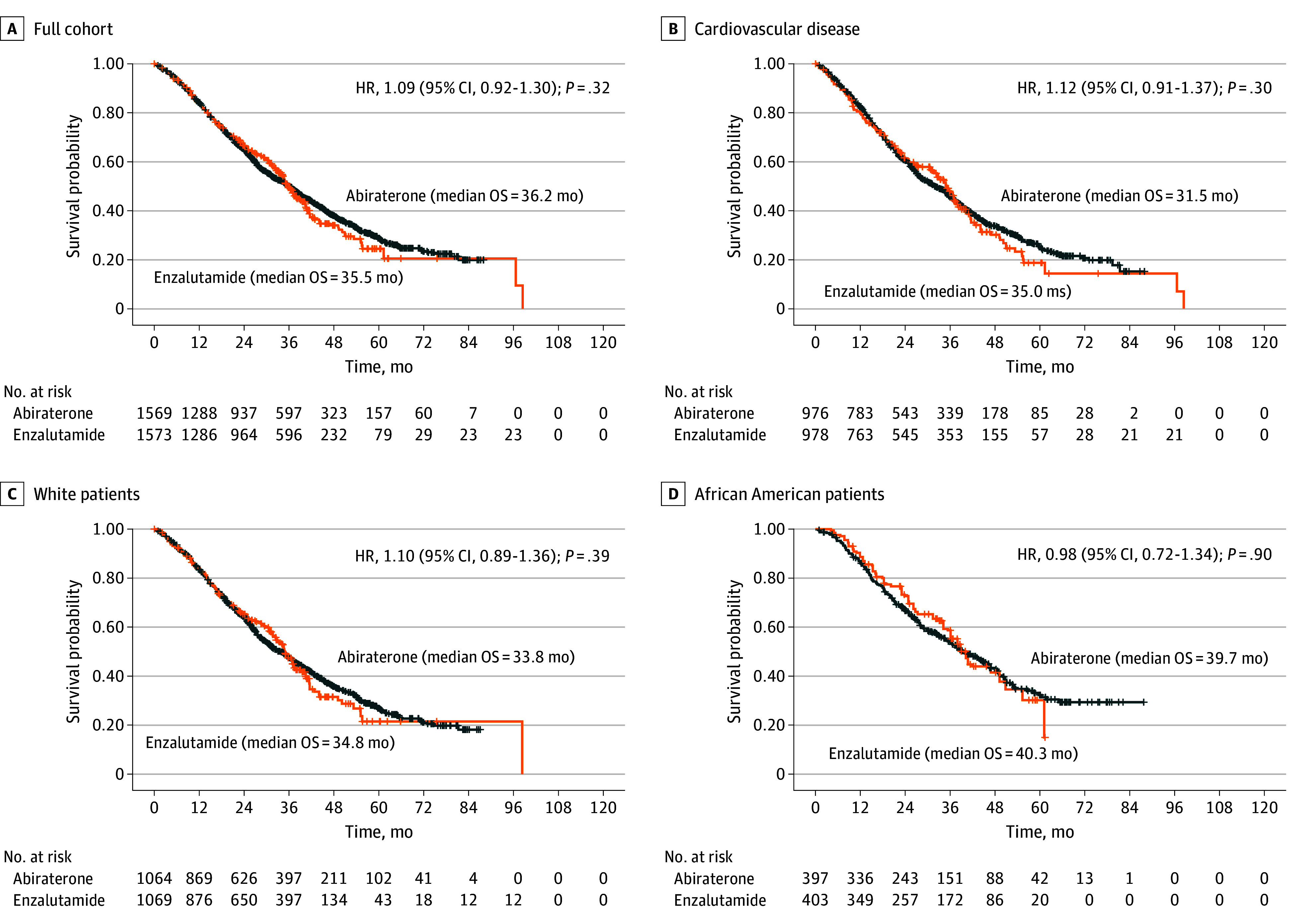
Comparing Median Overall Survival (OS) for Abiraterone vs Enzalutamide All 4 Kaplan-Meier analyses examine the difference in survival in abiraterone vs enzalutamide. There was neither a difference in OS in the full inverse probability weight–matched cohort (A) nor in those with cardiovascular diseases (B). Additionally, there was no OS difference between the 2 drugs in matched White (C) and African American (D) subgroups.

We performed multivariable IPW analyses and created 4 models incorporating various combinations of either numeric or continuous or categorical data inputs from the variables of interest (eTable 3 in Supplement 1). We also performed subgroup analyses in the White and African American races, and a subgroup of patients with cardiovascular diseases. There was no statistically significant difference in OS when comparing abiraterone and enzalutamide as first-line therapy for mHSPC in all 4 IPW model combinations. All subgroups of White patients, African American patients, and those with cardiovascular comorbidities did not show any statistically significant difference in OS as well. (Figures 3A-3D; eFigure 4 in Supplement 1).

### 1:1 Matched Cohort Result

**Figure 4.**
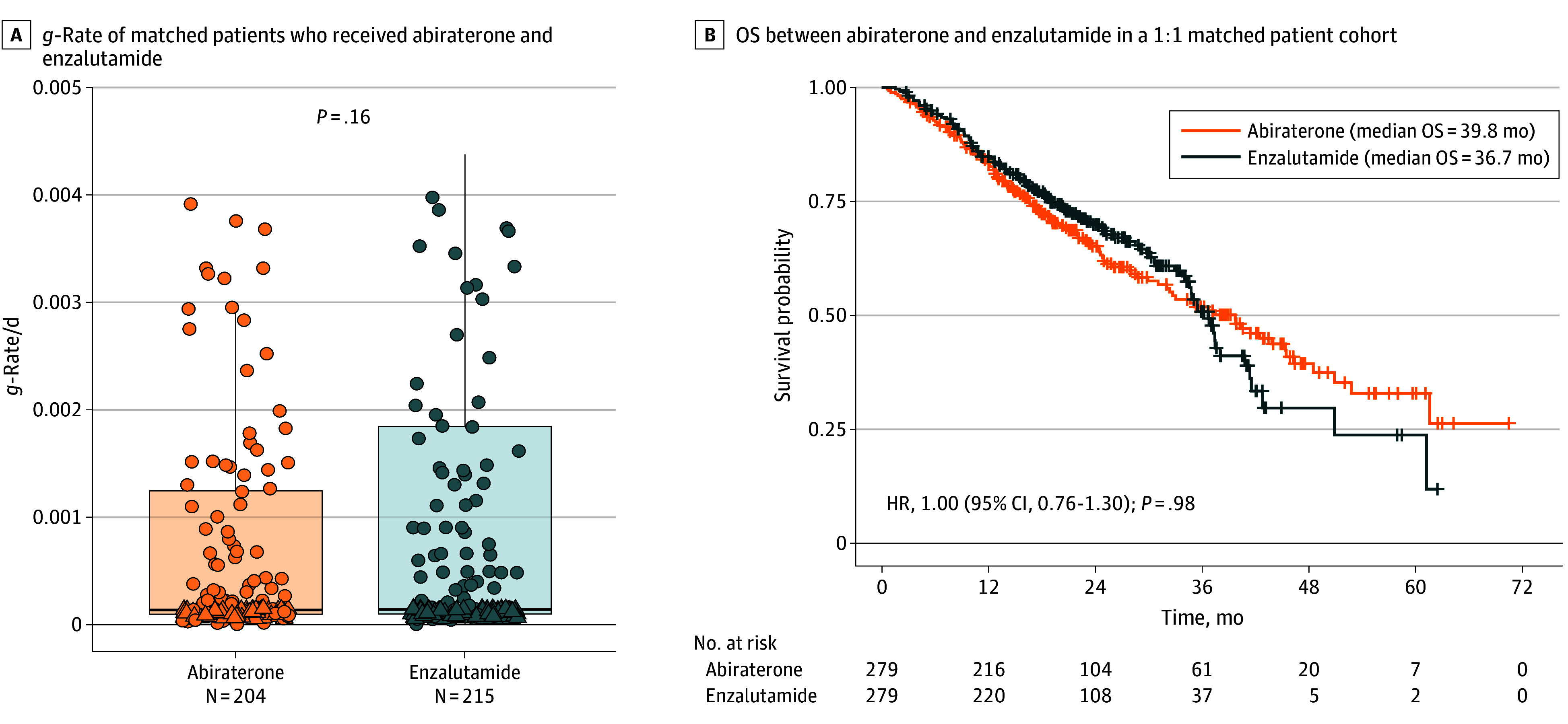
Comparing Median Overall Survival (OS) for Abiraterone vs Enzalutamide A, Growth rates (*g*-rates) of matched patients who received abiraterone and enzalutamide. Dots indicate individual data; box, IQR; line, median; and whiskers, data that fall within 1.5 times the IQR. Dots outside of whiskers indicate outliers. B, Kaplan-Meier analysis comparing OS between abiraterone and enzalutamide in a 1:1 matched patient cohort.

We identified a total of 279 patients for the abiraterone and the enzalutamide cohorts matched 1:1 on age (<70, 70-74, 75-79 or ≥80 years), race, drug start year (≤2018 or ≥2020), PSA at diagnosis (<50 or ≥50 ng/mL), CCI excluding cancer diagnosis (<5 or ≥5), and Gleason score (<8 or ≥8) (eTable 4 in Supplement 1). The median *g*-rate for the abiraterone cohort was 0.000134/d (DT, 5173 days), with a median OS of 39.8 (95% CI, 32.1-46.4) months at a median (IQR) follow-up time of 27.3 (15.3-40.8) months. Neither of these values were statistically different than the matched enzalutamide cohort’s median *g*-rate of 0.000139/d (DT, 4987 days), median (IQR) OS of 36.7 (34.6-41.4) months, and median (IQR) follow-up time of 31.1 (16.8-39.9) months (Figures 4A, 4B).

We further explored whether there is any difference between the 2 medications in a subgroup of patients who either had higher-grade disease (Gleason score ≥8; 316 patients) or higher-volume disease by starting PSA (≥50 ng/mL; 110 patients). As with the entire cohort, there was no statistical difference in median OS between the abiraterone and enzalutamide cohorts with higher Gleason grades (median OS, 40.5; 95% CI, 35.2-not reached [NR] vs 36.7; 95% CI, 34.3-NR) nor in those with higher PSA values (median OS, 33.1; 95% CI, 21.0-NR vs 27.4; 95% CI, 16.2-NR months) subgroups (eFigure 5 in Supplement 1). We also analyzed matched cohorts of White (203 patients in each cohort) and African American (63 patients in each cohort) veterans. For the White veterans, there was no difference in median OS between the 2 groups (median OS, 39.8; 95% CI, 28.7-50.9 vs 35.5; 95% CI, 33.8-37.5) (eFigure 6A in Supplement 1). For African American veterans, there was no significant difference in median OS for abiraterone vs enzalutamide, either (median OS, 37.5; 95% CI, 32.1-NR vs 50.9; 95% CI, 34.3-NR) (eFigure 6B in Supplement 1).

## Discussion

We present a clinical practice–based comparison of 1569 veterans treated with abiraterone or enzalutamide as first-line treatment for mHSPC, which includes 398 African American men, providing the most extensive data available regarding the safety and outcomes of these drugs in African American men. We noted similar *g* values and median OS for both cohorts at a median follow-up of 29 and 31 months for abiraterone and enzalutamide, respectively. To account for potential confounders, we performed 2 different analyses—IPW and 1:1 exact match—and conducted subgroup analyses. We did not observe a difference in median OS or *g*-rates in any of the matching and subgroup analyses. To our knowledge, this is the first direct comparison of abiraterone and enzalutamide for mHSPC in a clinical practice setting.

The difference in the number of patients receiving abiraterone compared with enzalutamide reflects the earlier approval of abiraterone in the mHSPC setting. Additionally, abiraterone became generic during this period of time and became the preferred medicine in the VHA. Interestingly, in our study, the prescribing patterns for abiraterone and enzalutamide did not differ based on comorbidities, except in the case of veterans with established liver impairment, who were more likely to receive enzalutamide. Prior studies have shown higher utilization of enzalutamide in older patients with more comorbid disease.^25^

Our *g*-rate method is ideal for clinical practice data analysis, as time is incorporated into the equations, rendering the assessment intervals irrelevant. This is an important attribute, given that clinical practice assessment intervals can vary significantly, unlike the rigid schedules of clinical trials. As a measure of outcomes, growth rates estimated using the *g*-rate method have been shown by us and the US FDA to highly correlate inversely with OS in numerous cancers treated with chemotherapy, targeted therapies, and immunotherapy.^21,26,27,28^ Additionally, we demonstrate that we can calculate *g*-rate as early as 3 months if there are at least 3 serial PSA values available 2 to 3 weeks apart within that timeframe.

In contrast to previous analyses that have found differences in overall survival between abiraterone and enzalutamide as first-line therapy in veterans with metastatic castration-resistant prostate cancer, we did not observe such a difference in this analysis.^15,25,29^ A possible explanation for this apparent discrepancy may be the significant contribution of LHRH agonists or antagonists to the activity of the combinations in mHSPC. In this earlier setting, the inclusion of an LHRH agonist or antagonist may have had a greater impact and likely reduced the relative contribution of abiraterone or enzalutamide to the combination, yielding comparable outcomes.

### Limitations and Strengths

All limitations applicable to an observational cohort study would also apply to our research, including the selection bias inherent in the initial choice of mHSPC therapy. Given data are obtained from electronic medical records, we did not have complete data regarding exact ADT start dates, patient performance status, volume of mHSPC, and site of metastases. The limitation in obtaining *g* values for certain patients comes from a lack of more than 2 PSA values, as in the clinical practice setting, there is no mandate to obtain PSA values. We performed 2 types of matching analyses to mitigate selection bias and improve confidence in the results. Given the large size of our data, we could perform an exact match to control for potential confounders and known prescribing patterns. This allowed us to compare 592 veterans with each other, and we did not identify differences in *g* values or OS between the abiraterone and enzalutamide groups. Variables like starting PSA values, Gleason scores, and CCI are numeric. In the exact 1:1 matching, we converted these variables to categorical ones to maximize the number of matches. Understanding matching on categorical variables can cause variance, and to include all patients in the matching analyses, we also performed IPW analyses. IPW allowed retention of these variables in their numeric format. However, small values, such as 0.01 in the continuous PSA values, are located at the tails of the propensity score distribution and could be assigned extreme weights, potentially increasing the variance and decreasing the balance between covariates.

Additionally, patients with missing values in the continuous variables are excluded from IPW analyses, whereas in categorical analyses, they can be assigned to a separate category of unknown. Understanding these statistical differences, we created 4 models that incorporated combinations of continuous and categorical data from variables of interest for IPW analyses. Due to known adverse events, there can be selection bias toward choosing enzalutamide in patients with already established cardiovascular comorbidities, given previously reported increased risk of cardiovascular events in patients treated with abiraterone.^30,31^ However, while there is a difference in the incidence of cardiac-related adverse events, hypertension, ischemic heart disease, and arrhythmia between the 2 drugs, it does not necessarily influence the incidence of cardiac arrests or deaths, as noted in a recent meta-analysis.^32^ Understanding the potential bias in one of the IPW models, we matched patients with cardiovascular comorbidities from the CCI—myocardial infarction, heart failure, peripheral vascular disease, and cerebrovascular disease—to assess the difference in OS between abiraterone and enzalutamide, and did not find any statistically significant OS difference between the 2 drugs. Although we did not have exact ADT start dates, our cohort was enriched for patients with the initiation of ARPIs within 6 months of their initial mHSPC diagnosis. We ensured that there was no PSA progression of disease before ARPI initiation, which is the enrollment criterion for the mHSPC registration trials for these drugs. The importance of clinical practice data are that there are no apparent limitations on patients’ performance status to get a medication as long as the physician deems it appropriate and it is more generalizable, as prostate cancer is more commonly a geriatric cancer, where it is more common to have limitations in performance status. We also attempted to obtain information on the sites of metastases and the volume of disease. A significant limitation to this is the small number of patients with visceral disease (93 of 765 patients [12.1%]) and lymph node-only disease (50 of 765 patients [6.5%]). Interestingly, patients with available information on sites or volume of disease appeared to have more aggressive disease, characterized by higher Gleason scores, higher PSA levels at the start of therapy, and more comorbidities, compared with the whole cohort; regardless, we did not observe any difference between abiraterone and enzalutamide, either based on volume of disease or sites of metastases.

As treatment for mHSPC evolves to include more triple therapy combinations, our data, combined with results from randomized trials such as ARASENS and PEACE-1, suggest that the choice of ARPI is unlikely to significantly impact survival, as noted by the absence of differences by volume or site of disease. Our study compared abiraterone with enzalutamide, 2 drugs with different mechanisms of action. It is unknown if medicines such as darolutamide or apalutamide would result in different outcomes. However, these agents have similar mechanisms of action to enzalutamide, and no prospective trials have shown significant differences between these agents.

## Conclusion

In this cohort study of 1569 veterans, abiraterone and enzalutamide performed similarly with similar *g*-rates estimated using PSA values and similar median OS. Both drugs have similar outcomes for African American and White veterans. It is important to note that enzalutamide is shown to be a more effective salvage treatment after progression while recieving abiraterone in metastatic castration-resistant prostate cancer than the reverse sequence.^15,33^ A substantial cost differential exists between the 2 drugs, as abiraterone is now available in generic form in the US, whereas enzalutamide is not. Given the comparable efficacy in PSA based *g*-rate and OS between abiraterone and enzalutamide as first-line therapy in mHSPC, our analysis supports considering cost, adverse event profile, patient preference, drug interactions with other medications, and optimal drug sequencing when determining initial therapy for mHSPC.

## Notes

Supplement 1. eTable 1. Tumor Growth Rate Fits eTable 2. Comparison of Full Cohort and Subcohort With Information on Sites of Metastases eTable 3. Inverse Probability Weight Models eTable 4. Baseline Characteristics of the 1:1 Matched Cohort eFigure 1. *g*-Rate at 3 and 6 Months eFigure 2. KM Analyses Based on Volume of Disease eFigure 3. KM Analyses Based on Sites of Metastases eFigure 4. IPW Model Explorations eFigure 5. Matched Cohort KM Analyses for Gleason 8 or Greater and PSA 50 or Greater eFigure 6. Matched Cohort KM Analyses for Race

Supplement 2. Data Sharing Statement
