# Supplementary material for: Abiraterone vs Enzalutamide Among US Veterans With Metastatic Hormone-Sensitive Prostate Cancer
Source: JAMA Netw Open. 2025 Nov 4;8(11):e2540730. doi: 10.1001/jamanetworkopen.2025.40730 (PMC12587203; doi:10.1001/jamanetworkopen.2025.40730)
Supplement: Supplement 1. — eTable 1. Tumor Growth Rate Fits eTable 2. Comparison of Full Cohort and Subcohort With Information on Sites of Metastases eTable 3. Inverse Probability Weight Models eTable 4. Baseline Characteristics of the 1:1 Matched Cohort eFigure 1. g-Rate at 3 and 6 Months eFigure 2. KM Analyses Based on Volume of Disease eFigure 3. KM Analyses Based on Sites of Metastases eFigure 4. IPW Model Explorations eFigure 5. Matched Cohort KM Analyses for Gleason 8 or Greater and PSA 50 or Greater eFigure 6. Matched Cohort KM Analyses for Race [file jamanetwopen-e2540730-s001.pdf]

Supplemental Online Content

Leuva H, Zhou M, Teply BA, et al. Abiraterone vs enzalutamide among US veterans with metastatic hormone-sensitive prostate cancer. *JAMA Netw Open*. 2025;8(11):e2540730. doi:10.1001/jamanetworkopen.2025.40730

eTable 1. Tumor Growth Rate Fits
eTable 2. Comparison of Full Cohort and Subcohort With Information on Sites of Metastases
eTable 3. Inverse Probability Weight Models
eTable 4. Baseline Characteristics of the 1:1 Matched Cohort
eFigure 1. *g*-Rate at 3 and 6 Months
eFigure 2. KM Analyses Based on Volume of Disease
eFigure 3. KM Analyses Based on Sites of Metastases
eFigure 4. IPW Model Explorations
eFigure 5. Matched Cohort KM Analyses for Gleason 8 or Greater and PSA 50 or Greater
eFigure 6. Matched Cohort KM Analyses for Race

This supplemental material has been provided by the authors to give readers additional information about their work.

© 2025 Leuva H et al. *JAMA Network Open*.

eTable 1. Tumor Growth Rate Fits						
N (%)	3 Months		6 Months		All	
	ABI	ENZA	ABI	ENZA	ABI	ENZA
**Calculable *g*-rate**						
	720(57)	155(50)	893(70)	204(66)	979 (78)	238(77)
**Model fit break down**						
dx	585 (47)	128 (41)	608 (48)	142 (46)	461 (37)	116 (37)
gdphi	5 (<1)	0 (<1)	32 (3)	3 (<1)	171 (14)	29 (9)
gd	80 (6)	12 (4)	168 (13)	36 (12)	185 (15)	49 (16)
gx	50 (4)	15 (5)	85 (7)	23 (7)	162 (13)	44 (14)
not fit	160 (13)	36 (12)	106 (8)	25 (8)	69 (5)	14 (5)
**Reason for non-calculable *g*-rate**						
only 1 point	285 (23)	97 (31)	208 (17)	66 (21)	186 (15)	54 (17)
2 points < 20%	41 (3)	12 (4)	28 (2)	12 (4)	21 (2)	4 (1)
no measurement data	52 (4)	11 (4)	23 (2)	4 (1)	3 (<1)	1 (<1)

© 2025 Leuva H et al. *JAMA Network Open*.

eTable 2. Comparison of Full Cohort and Subcohort With Information on Sites of Metastases			
	Full Cohort (N=1,569)	Sub cohort (N=765)	p value
Age, yrs (median, IQR)	73 (69-79)	74 (69-79)	0.61
Age group (N, %)			
<70	443 (28.2)	220 (28.8)	0.80
70-74	433 (27.6)	222 (29.0)	
75-79	324 (20.7)	147 (19.2)	
≥80	369 (23.5)	176 (23.0)	
Race (N, %)			
Caucasian	1064 (67.8)	513 (67.1)	0.60
African American	398 (25.4)	206 (26.9)	
Other*/Unknown	107 (6.8)	46 (6.0)	
Ethnicity (N, %)			
Hispanic	66 (4.2)	31 (4.1)	0.77
Not Hispanic	1397 (89.0)	688 (89.9)	
Unknown	106 (6.8)	46 (6.0)	
Rurality (N, %)			
Urban	1132 (72.1)	560 (73.2)	0.44
Rural	393 (25.0)	178 (23.3)	
Unknown	44 (2.8)	27 (3.5)	
PSA at diagnosis (median, IQR)	37 (7-209)	78 (20-340)	<0.0001*
PSA at treatment start (median, IQR)	8 (1-46)	11 (2-56)	0.0029*
PSA at treatment start group (N, %)			
<50	1196 (76.2)	553 (72.3)	0.04*
≥50	373 (23.8)	212 (27.7)	
Gleason score (N, %)			
<8	206 (13.1)	55 (7.2)	<0.0001*
≥8	841 (53.6)	451 (59.0)	
Unknown	522 (33.3)	259 (33.9)	
Start year of ABI/ENZA (N, %)			
Prior to 2018	150 (9.6)	41 (5.4)	0.0016*
2018-2020	923 (58.8)	457 (59.7)	
2021 to present	496 (31.6)	267 (34.9)	

© 2025 Leuva H et al. *JAMA Network Open*.

eTable 2 continued			
	Full Cohort (N=1,569)	Sub cohort (N=765)	P value
Treatment duration, days (median, IQR)	372 (163-706)	386 (171-712)	0.58
Other prior treatment (N, %)			
Surgery	127 (8.1)	51 (6.7)	0.26
Radiotherapy	731 (46.6)	390 (51.0)	0.05
Charlson Comorbidity Index (N, %) w/o cancer			
<5	1167 (74.4)	525 (68.6)	0.0040*
≥5	402 (25.6)	240 (31.4)	
Comorbidities, detailed (N, % of patients with the condition)			
Myocardial infarction	251 (16.0)	137 (17.9)	0.27
Heart failure	422 (26.9)	235 (30.7)	0.06
Peripheral vascular disease	623 (39.7)	342 (44.7)	0.02*
Cerebrovascular disease	407 (25.9)	224 (29.3)	0.09
Dementia	117 (7.5)	63 (8.2)	0.56
Chronic pulmonary disease	592 (37.7)	318 (41.6)	0.08
Rheumatic disease	195 (12.4)	111 (14.5)	0.18
Peptic ulcer disease	61 (3.9)	36 (4.7)	0.41
Mild liver disease	269 (17.1)	137 (13.9)	0.69
Diabetes with/without complications	654 (41.7)	349 (45.6)	0.08
Paraplegia and hemiplegia	83 (5.3)	53 (6.9)	0.14
Renal disease	503 (32.1)	273 (35.7)	0.09
Moderate or severe liver disease	15 (1.0)	8 (1.0)	0.83
HIV	11 (0.7)	5 (0.7)	0.99
Median Follow-up (months) Oct 2024	29.1	28.7	0.42

© 2025 Leuva H et al. *JAMA Network Open*.

eTable 3. Inverse Probability Weight Models						
	Common variables	Starting PSA	Start year	CCI	HR (ABI as ref)	p-value
Full Cohort						
Model 1	Age, race	C	C	C	1.093 (0.918-1.301)	0.32
Model 2		C	C	C*	1.080 (0.908-1.284)	0.38
Model 3		N	C	N	1.049 (0.883-1.247)	0.58
Model 4		N	C	C*	1.057 (0.890-1.254)	0.53
Subgroups						
Caucasian	Age	C	C	C	1.098 (0.887-1.359)	0.39
African American					0.980 (0.715-1.343)	0.90
With Cardiovascular diseases	Age, race	C	C	C	1.117 (0.907-1.374)	0.30
Abbreviations: C- categorical; CCI-Charlson comorbidity index; C*- matched on 4 comorbidities only (any of the four or none): myocardial infarction, heart failure, peripheral vascular disease, cerebrovascular disease; N- numeric						

© 2025 Leuva H et al. *JAMA Network Open*.

eTable 4. Baseline Characteristics of the 1:1 Matched Cohort						
	Abiraterone **Full** (N=1,258)	Enzalutamide **Full** (N=311)	p value	Abiraterone **Matched** (N=279)	Enzalutamide **Matched** (N=279)	p value
Age, yrs (median, IQR)	73 (69-79)	74 (69-79)	0.29	74 (69-79)	74 (69-79)	0.97
Age group (N, %)						
<70	364 (28.9)	79 (25.4)	0.61	72 (25.8)	72 (25.8)	matched
70-74	341 (27.1)	92 (29.6)		77 (27.6)	77 (27.6)	
75-79	257 (20.4)	67 (21.5)		61 (21.9)	61 (21.9)	
≥80	296 (23.5)	73 (23.5)		69 (24.7)	69 (24.7)	
Race (N, %)						
Caucasian	857 (68.1)	207 (66.6)	0.74	203 (72.8)	203 (72.8)	matched
African American	314 (25.0)	84 (27.0)		63 (22.6)	63 (22.6)	
Other*/Unknown	87 (6.9)	20 (6.4)		13 (4.7)	13 (4.7)	
Ethnicity (N, %)						
Hispanic	55 (4.4)	11 (3.5)	0.50	9 (3.2)	11 (3.9)	0.90
Not Hispanic	1122 (89.2)	275 (88.4)		249 (89.2)	247 (88.5)	
Unknown	81 (6.4)	25 (8.0)		21 (7.5)	21 (7.5)	
Rurality (N, %)						
Urban	912 (72.5)	220 (70.7)	0.74	204 (73.1)	194 (69.5)	0.47
Rural	310 (24.6)	83 (26.7)		67 (24.0)	79 (28.3)	
Unknown	36 (2.9)	8 (2.6)		8 (2.9)	6 (2.2)	
PSA at diagnosis (median, IQR)	39 (7-203)	36 (8-242)	0.89	30 (6-125)	30 (7-188)	0.52
PSA at treatment start (median, IQR)	8 (1-46)	8 (1-44)	0.31	7 (2-34)	7 (0.70-36)	0.32
PSA at treatment start group (N, %)						
<50	958 (76.2)	238 (76.5)	0.95	224 (80.3)	224 (80.3)	matched
≥50	300 (23.8)	73 (23.5)		55 (19.7)	55 (19.7)	
Gleason score (N, %)						
<8	164 (14.0)	42 (13.2)	0.83	32 (11.5)	32 (11.5)	matched
≥8	671 (53.3)	170 (55.0)		158 (56.6)	158 (56.6)	
Unknown	423 (33.6)	99 (31.8)		89 (31.9)	89 (31.9)	
Start year of ABI/ENZA (N, %)						
Prior to 2018	136 (10.8)	14 (4.5)	<0.0001*	10 (3.6)	10 (3.6)	matched
2018-2020	784 (62.3)	139 (44.7)		133 (47.7)	133 (47.7)	
2021 to present	338 (26.9)	158 (50.8)		136 (48.7)	136 (48.7)	

© 2025 Leuva H et al. *JAMA Network Open*.

eTable 4 - continued						
	Abiraterone **Full** (N=1,258)	Enzalutamide **Full** (N=311)	p value	Abiraterone **Matched** (N=279)	Enzalutamide **Matched** (N=279)	p value
Treatment duration, days (median, IQR)	360 (162-720)	416 (171-680)	0.97	360 (170-653)	442 (174-705)	0.43
Other prior treatment (N, %)						
Surgery	98 (7.8)	29 (9.3)	0.44	26 (9.3)	24 (8.6)	0.88
Radiotherapy	602 (47.9)	129 (41.5)	0.05	120 (43.0)	116 (41.6)	0.80
Charlson Comorbidity Index (N, %) w/o cancer						
<5	938 (74.6)	229 (73.6)	0.79	211 (75.6)	211 (75.6)	matched
≥5	320 (25.4)	82 (26.4)		68 (24.4)	68 (24.4)	
Comorbidities, detailed (N, % of patients with the condition)						
Myocardial infarction	202 (16.1)	49 (15.8)	0.97	36 (12.9)	42 (15.1)	0.54
Heart failure	334 (26.6)	88 (28.3)	0.58	64 (22.9)	78 (28.0)	0.21
Peripheral vascular disease	506 (40.2)	117 (37.6)	0.44	112 (40.1)	102 (36.6)	0.43
Cerebrovascular disease	336 (26.7)	71 (22.8)	0.19	73 (26.2)	65 (23.3)	0.49
Dementia	97 (7.7)	20 (6.4)	0.52	23 (8.2)	19 (6.8)	0.63
Chronic pulmonary disease	466 (37.0)	126 (40.5)	0.29	100 (35.8)	115 (41.2)	0.22
Rheumatic disease	157 (12.5)	38 (12.2)	0.98	34 (12.2)	33 (11.8)	0.99
Peptic ulcer disease	47 (3.7)	14 (4.5)	0.64	10 (3.6)	11 (3.9)	0.99
Mild liver disease	201 (16.0)	68 (21.9)	0.0172*	50 (17.9)	59 (21.1)	0.39
Diabetes with/without complications	510 (40.5)	144 (46.3)	0.07	101 (36.2)	128 (45.9)	0.03*
Paraplegia and hemiplegia	73 (5.8)	10 (3.2)	0.09	11 (3.9)	7 (2.5)	0.47
Renal disease	396 (31.5)	107 (34.4)	0.36	86 (30.8)	93 (33.3)	0.59
Moderate or severe liver disease	11 (0.9)	4 (1.3)	0.51	3 (1.1)	3 (1.1)	1.00
HIV	11 (0.9)	0 (0)	0.14	3 (1.1)	0 (0)	0.25
Median Follow-up (months) Apr 2023	23.2	20.4	<0.0001	19.5	21.0	0.96
Median Follow-up (months) Oct 2024	28.7	30.8	0.12	27.3	31.1	0.58

© 2025 Leuva H et al. *JAMA Network Open*.

eFigure 1. *g*-Rate at 3 and 6 Months

g-rate using only first 3 Months PSA after starting therapy

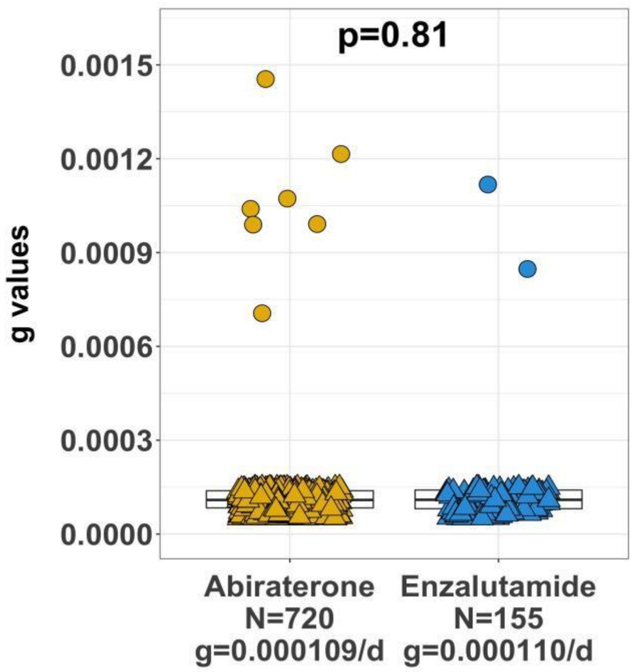

g-rate using only first 6 Months PSA after starting therapy

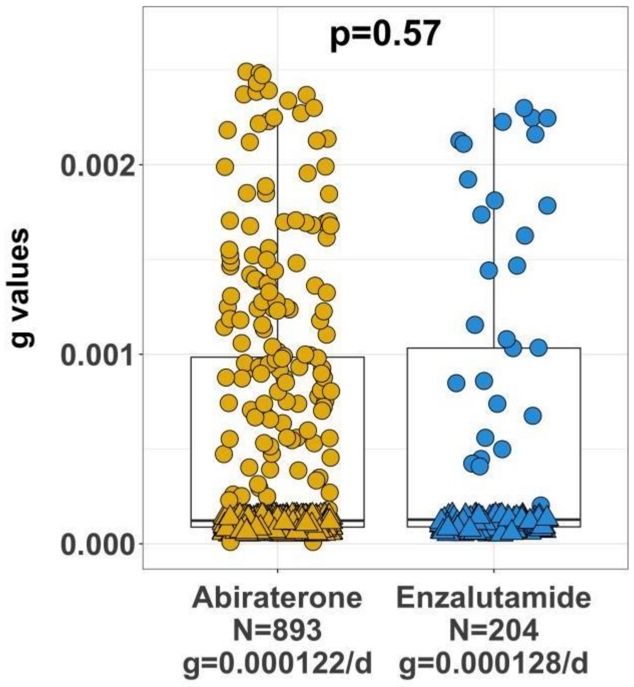

© 2025 Leuva H et al. *JAMA Network Open*.

eFigure 2. KM Analyses Based on Volume of Disease

2A - High Volume

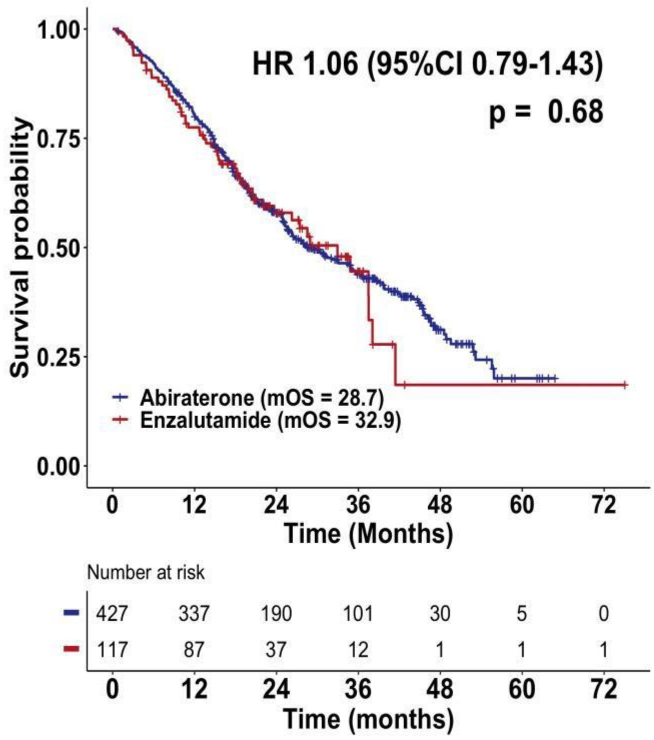

2B- Low Volume

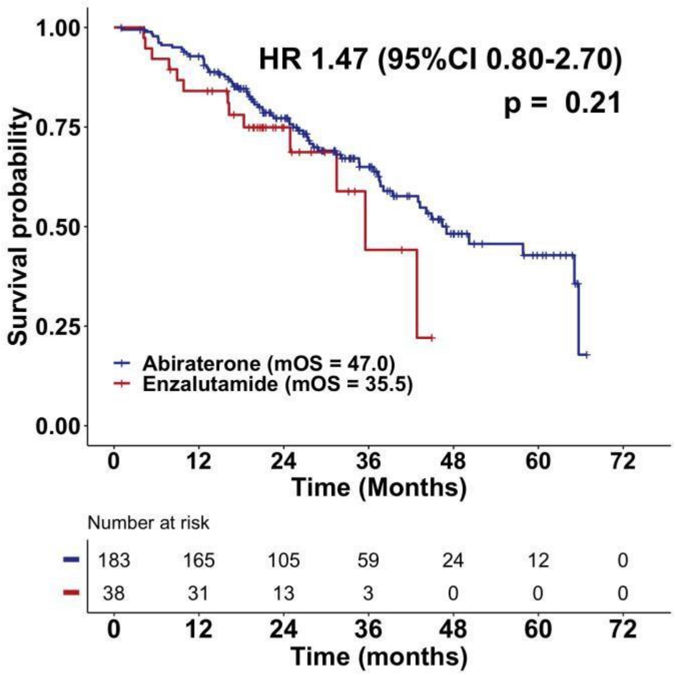

© 2025 Leuva H et al. *JAMA Network Open*.

eFigure 3. KM Analyses Based on Sites of Metastases

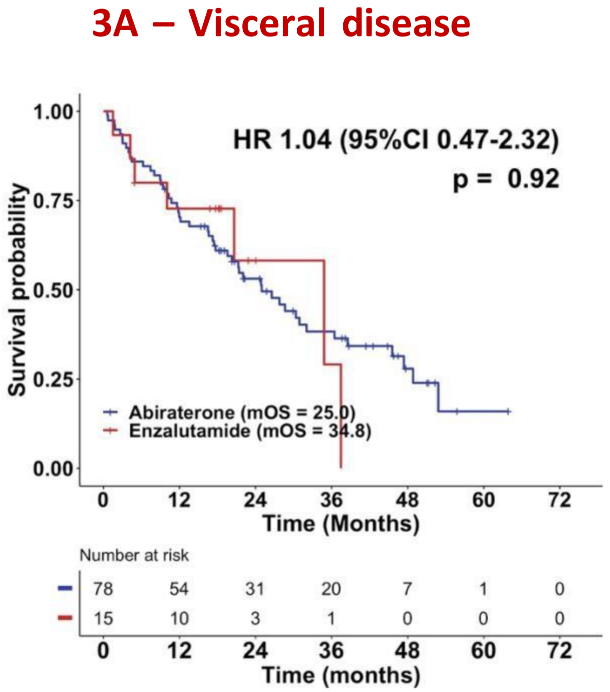

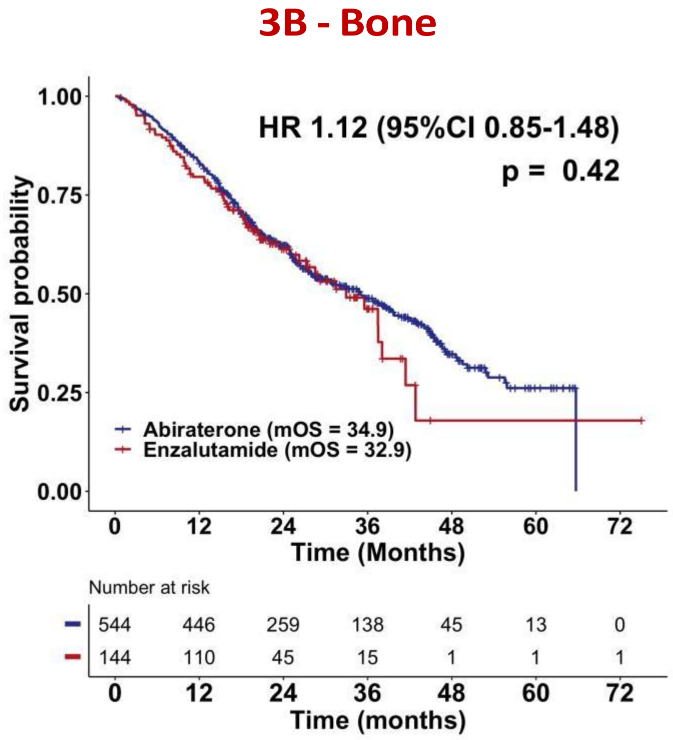

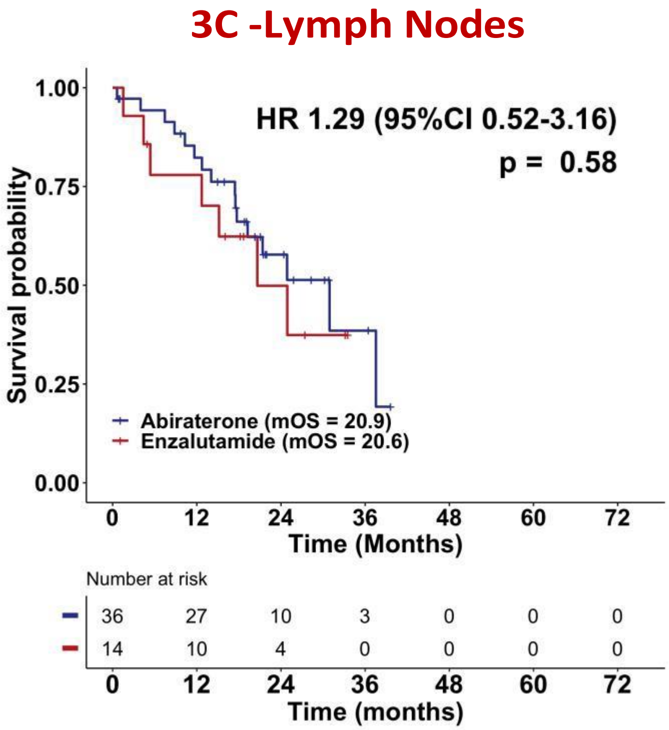

© 2025 Leuva H et al. *JAMA Network Open*.

eFigure 4. IPW Model Explorations

Full cohort – IPW Model 2

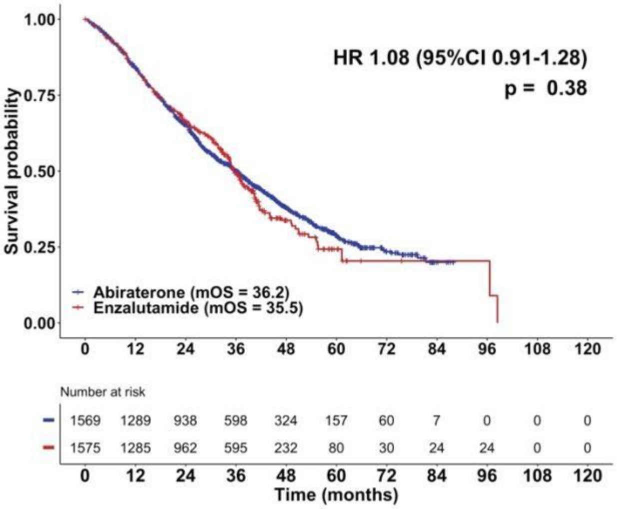

Full cohort – IPW Model 3

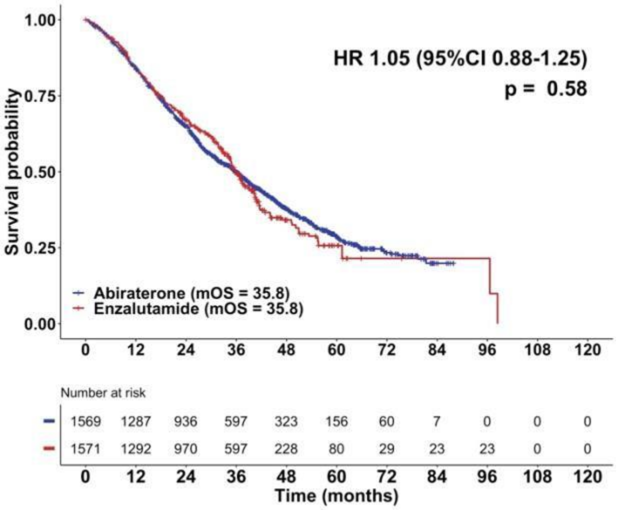

Full cohort – IPW Model 4

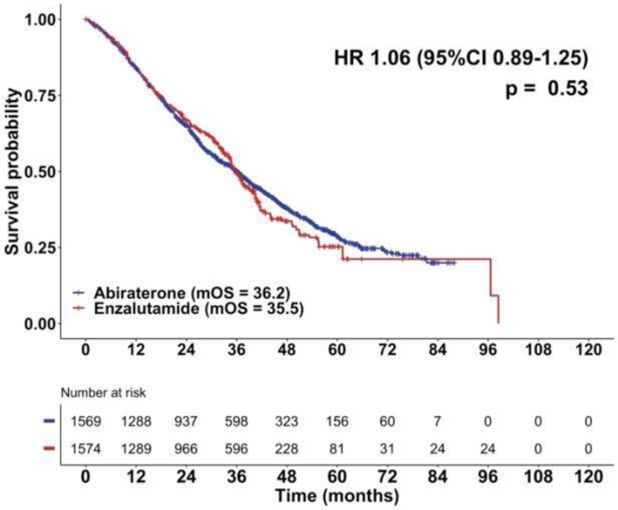

© 2025 Leuva H et al. *JAMA Network*

eFigure 5. Matched Cohort KM Analyses for Gleason 8 or Greater and PSA 50 or Greater

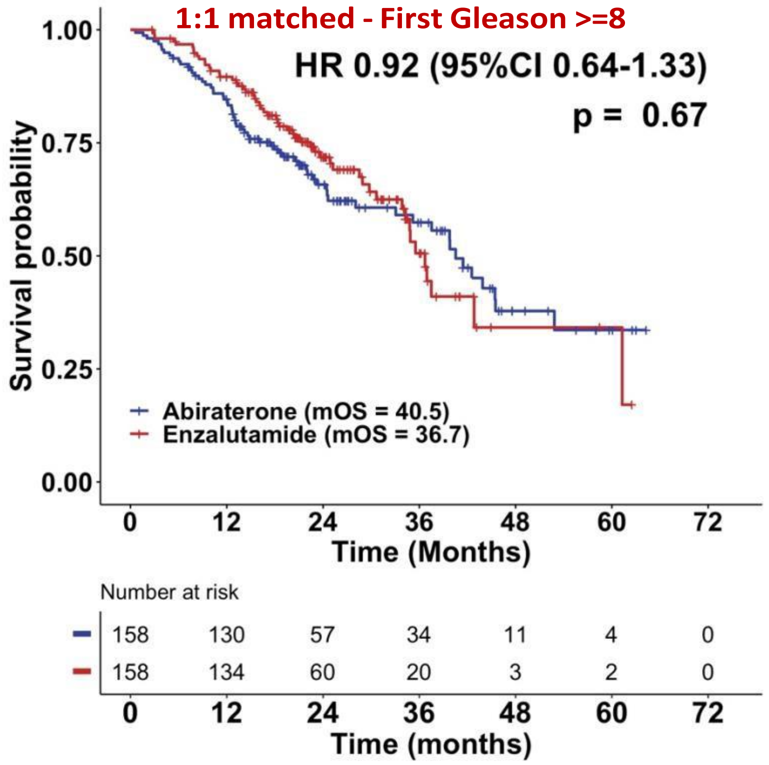

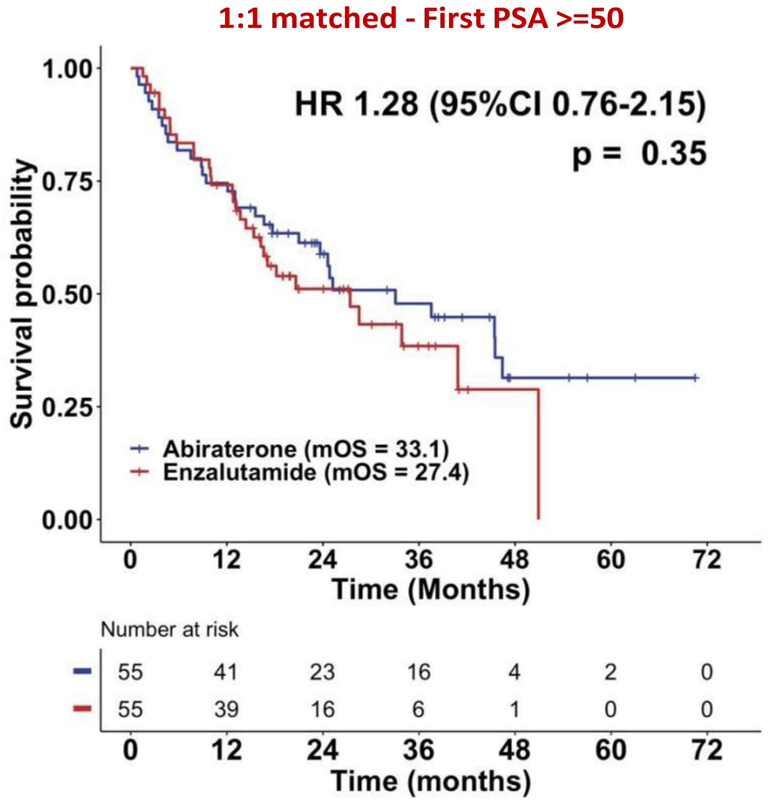

© 2025 Leuva H et al. *JAMA Network Open*.

eFigure 6. Matched Cohort KM Analyses for Race

6A - 1:1 matched -Caucasians

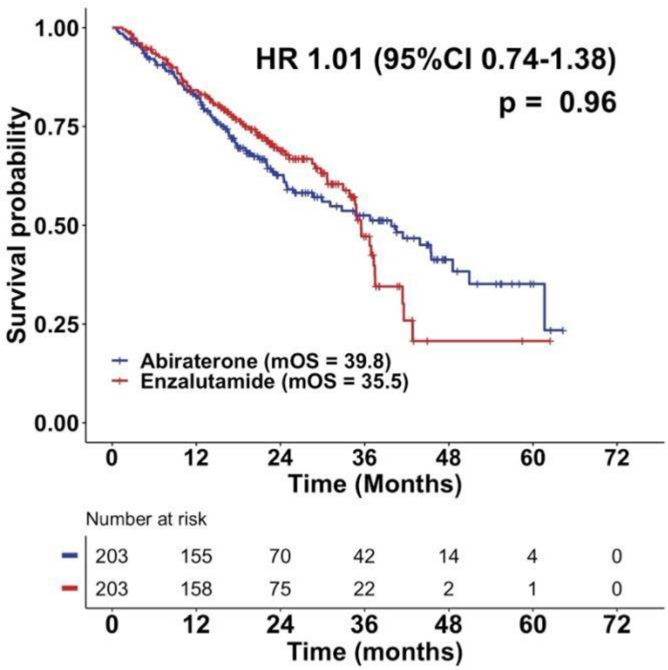

6B - 1:1 matched - African Americans

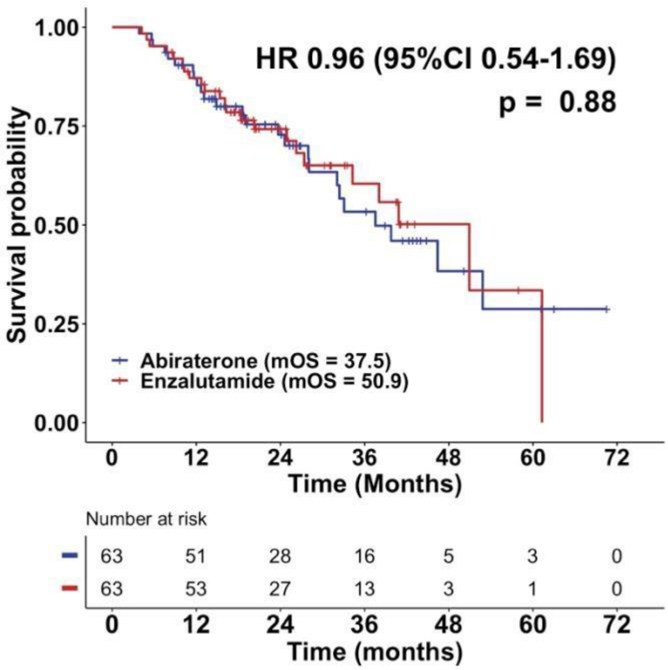

© 2025 Leuva H et al. *JAMA Network Open*.
