# Supplementary material for: Abiraterone vs Enzalutamide Among US Veterans With Metastatic Hormone-Sensitive Prostate Cancer
Source: JAMA Netw Open. 2025 Nov 4;8(11):e2540730. doi: 10.1001/jamanetworkopen.2025.40730 (PMC12587203; doi:10.1001/jamanetworkopen.2025.40730)
Supplement: Supplement 2. — Data Sharing Statement [file jamanetwopen-e2540730-s002.pdf]

Data Sharing Statement

Leuva. Abiraterone vs Enzalutamide Among US Veterans With Metastatic Hormone-Sensitive Prostate Cancer (mHSPC). *JAMA Netw Open*. Published November 03, 2025. doi:10.1001/jamanetworkopen.2025.40730

Data
Data available: No

Additional Information
Explanation for why data not available: VA data requires permission to use before sharing, so we might not be able to make the dataset public. Based on requests, we should be able to provide anonymized data.
